# ADHD among young adults born at extremely low birth weight: the role of fluid intelligence in childhood

**DOI:** 10.3389/fpsyg.2014.00446

**Published:** 2014-05-19

**Authors:** Ayelet Lahat, Ryan J. Van Lieshout, Saroj Saigal, Michael H. Boyle, Louis A. Schmidt

**Affiliations:** ^1^Department of Psychology, Neuroscience and Behaviour, McMaster UniversityHamilton, ON, Canada; ^2^Department of Psychiatry and Behavioural Neurosciences, McMaster UniversityHamilton, ON, Canada; ^3^Department of Pediatrics, McMaster UniversityHamilton, ON, Canada

**Keywords:** executive function, fluid intelligence, ADHD, ELBW, longitudinal

## Abstract

Poor executive function (EF) has been linked to attention-deficit/hyperactivity disorder (ADHD). Children born at extremely low birth weight (ELBW; <1000 g) have been found to show both poor EF, as well as elevated levels of symptoms of ADHD. In the present study, we examined whether fluid intelligence moderates the link between birth weight and later ADHD symptoms by prospectively following a cohort of 179 survivors who were born at ELBW. When participants were 8 years-old, they were matched with 145 normal birth weight (NBW; ≥2500 g) control participants. At age 8, fluid intelligence was measured, and during young adulthood (ages 22–26), participants' self-reported levels of ADHD symptoms were examined. We found that ELBW survivors, who also showed poor fluid intelligence, had the highest rates of ADHD symptoms, and particularly, symptoms of inattention. These findings point to the importance of examining developmental trajectories that contribute to risk for psychopathology in those exposed to intrauterine adversity.

## Introduction

Extremely low birth weight (ELBW; <1000 g) survivors are among the tiniest and most vulnerable babies. Compared to individuals born at normal birth weight (NBW; ≥2500 g), those born at very low birth weight (VLBW; <1500 g) and smaller have been found to be at increased risk for later psychopathology, including Attention Deficit/Hyperactivity Disorder (ADHD; Szatmari et al., 1990, 1993; Ross et al., 1991; Botting et al., 1997; Whitaker et al., 1997; Taylor et al., 2000; Bhutta et al., 2002; Elgen et al., 2002; Foulder-Hughes and Cooke, 2003; Indredavik et al., 2004; Strang-Karlsson et al., 2008; Hack et al., 2009; Johnson et al., 2010; Johnson and Marlow, 2011). However, not all ELBW survivors develop ADHD, and very little is known about the developmental trajectories that lead to risk and resilience among these individuals. Accordingly, the aim of the present study was to examine the role of executive function (EF), and specifically fluid intelligence, which may serve as a putative mechanism underlying variation in ADHD risk among individuals born at ELBW.

It is important to point out that although not all low birth weight babies are born prematurely, most babies born ELBW and VLBW are. Premature birth may be associated with a greater risk for symptoms of inattention than hyperactivity/impulsivity, and some studies have reported higher rates of the inattentive subtype of ADHD compared with hyperactive/impulsive subtype in ELBW and VLBW children (Botting et al., 1997; Indredavik et al., 2004; Hack et al., 2009; Johnson et al., 2010). Indeed, some have proposed (Strang-Karlsson et al., 2008) that the ADHD of preterm children is more “pure,” as it is characterized by less hyperactivity in relation to inattention, as well as by a more even sex distribution, and it is less frequently accompanied by comorbid disorders (Szatmari et al., 1993; Botting et al., 1997; Elgen et al., 2002; Indredavik et al., 2004). These findings have led some (Szatmari et al., 1990; Hille et al., 2001) to suggest that premature children are susceptible to a more biologically determined form of attention deficit associated with impaired brain growth (Peterson et al., 2000, 2003; Rushe et al., 2001; Kapellou et al., 2006).

For example, Indredavik et al. (2005) found that ADHD symptoms were associated with reduction in white matter volumes and thinning of the corpus callosum in VLBW adolescents. This correlation between symptoms and white matter volume was due primarily to a specific association with inattention scores. In a separate study (Skranes et al., 2007), inattention, but not hyperactivity scores, were associated with fractional anisotropy measurements of white matter in VLBW adolescents. Such white matter abnormalities are associated with difficulties in EF (Edgin et al., 2008), the control over thought and action in situations that require problem solving (Zelazo et al., 2008). Thus, impairments in the underlying cognitive mechanisms that are associated with these structural brain differences, such as EF, could have important implications for later developmental outcomes.

EF also has been referred to as fluid intelligence (Blair, 2006). Although the relation between EF and fluid intelligence has been debated (Birney et al., 2006; Burgess et al., 2006; Garlick and Sejnowski, 2006; Heitz et al., 2006), both entail cognitive processing not necessarily associated with any specific content domain, and involve the active or effortful maintenance of information in working memory for the purposes of planning and performing goal-directed behavior (Kane and Engle, 2002). Since domain general indicators of cognitive abilities involve functions such as information maintenance, attention shifting, and resistance to interference—measures of fluid intelligence have demonstrated significant associations with performance on measures of general intelligence (Embretson, 1995; Engle et al., 1999). However, there is also evidence for a dissociation between fluid intelligence and general intelligence (see Blair, 2006, for a review), namely, fluid intelligence seems to be a specific subset of more global cognitive abilities (Séguin and Zelazo, 2005).

Impairments in EF/fluid intelligence, particularly in the domains of response inhibition, planning, vigilance, and working memory have been associated with ADHD (see Pennington and Ozonoff, 1996; Willcutt et al., 2005). Studies examining the association between EF and ADHD suggest that poor EF is primarily associated with inattentive symptoms of ADHD rather than hyperactivity or impulsivity (Chhabildas et al., 2001; Nigg et al., 2005; Willcutt et al., 2005). Given that inattentive symptoms of ADHD are more prevalent among individuals born prematurely, it is likely that fluid intelligence plays a role in the development of ADHD among individuals born at ELBW. Indeed, Nadeau et al. (2001) observed that general cognitive ability mediated the relation between extreme preterm birth and hyperactivity, whereas the relation between extreme preterm birth and inattention was mediated specifically by working memory, a specific type of EF.

Children and adolescents who were born preterm have been found to have poorer EF abilities than those born at term (Anderson and Doyle, 2004; Böhm et al., 2007; Luu et al., 2011; Baron et al., 2012). For example, compared to term controls, adolescents born preterm showed deficits in EF abilities, including verbal fluency, inhibition, cognitive flexibility, planning/organization, and working memory, as well as poorer verbal and visuospatial memory (Luu et al., 2011). Böhm et al. (2007) reported that NBW controls surpassed VLBW children on EF, even after controlling for IQ. In another study, at 3 years of age, children born at ELBW performed more poorly than term-born age-mates on working memory and inhibition tasks and had the highest percentage of incomplete performance on a continuous performance test (Baron et al., 2012). Finally, in a different report comparing 8–9 years old ELBW survivors to their NBW peers (Anderson and Doyle, 2004), EF was reduced in the ELBW group.

Given that not all ELBW survivors go on to develop ADHD, and since poor EF is associated with both ELBW and ADHD, we examined here the moderating role of fluid intelligence in understanding the relation between ELBW and symptoms of ADHD. Thus, we conducted a prospective longitudinal study, which included three different time points: (1) birth, (2) middle childhood (age 8), and (3) young adulthood (ages 22–26). This allowed us to examine a developmental trajectory leading to developmental outcomes. We were interested in predicting from birth into young adulthood, which was our endpoint visit.

The cohort of ELBW survivors was followed-up at age 8 and again at 22–26 years of age. A control sample matched on age, sex, and SES was recruited at 8 years of age. During the 8 years visit, participants completed Raven's Colored Progressive Matrices Test (RCPM; Raven, 1983), a measure of fluid intelligence (Blair, 2006) and the Wechsler Intelligence Scale for Children (WISC-R; Wechsler, 1974), measuring general intelligence. As young adults, participants completed the ADHD Rating Scale (Barkley and Murphy, 1998), and the Young Adult Self Report (YASR; Achenbach, 1997). We expected that fluid intelligence would moderate the link between birth weight group and symptoms of ADHD, such that among participants with poor fluid intelligence, ELBW survivors would have the greatest level of ADHD symptoms. Given that ELBW and EF are both linked to the inattentive sub type of ADHD, we expected to find an interaction between birth weight group and fluid intelligence, particularly for inattentive symptoms of ADHD.

## Methods

### Participants

This study followed-up a cohort of 397 predominantly Caucasian infants who were born at ELBW (501–1000 g) between 1977 and 1982 to residents of a geographically defined region in central-west Ontario, Canada. Follow-up assessments were conducted when participants were 8- (childhood) and 22–26- (young adulthood) years old. Of the original 397 infants, 179 (45%) survived to hospital discharge from the NICU. There were 13 late deaths, and 166 survived to young adulthood.

During the young adult visit, data were collected on 142 of the 166 (86%) survivors. Reasons for missing data include loss to follow-up (*N* = 9) and refusal (*N* = 8). An additional seven participants had neurosensory impairments (cerebral palsy, blindness, deafness, mental retardation, and microcephaly) and could not complete the assessments. Of these 142, a total of 125 had complete data on the measures collected at the 8-years visit.

The NBW control group was identified and recruited when they and the ELBW cohort were 8 years old. This group comprised a sample of 145 children born at term according to maternal report, between 1977 and 1981. The control sample was selected from class lists provided by local school boards and group-matched with the ELBW cohort on child age, sex, and socioeconomic status (Saigal et al., 1991). Data were collected on 133 of the 145 control participants. Reasons for missing data include loss to follow-up (*N* = 5) and refusal (*N* = 7). All 133 participants had complete data on the measures collected at the 8-years visit.

Data were examined for outliers and participants with more than ±2 SDs from the mean were removed from all analyses. These outliers were removed as they can affect the mean dramatically and not represent the majority of the group. This resulted in four ELBW and five NBW participants being dropped from the analyses, and thus the final sample included 121 ELBWs and 128 NBWs.

### Measures

#### Raven's Colored Progressive Matrices (RCPM; Raven, 1983)

The RCPM was administered when children were 8 years of age. This is a non-verbal measure of fluid intelligence in which the participant is shown colored illustrations with one part missing. The participant is asked to identify and select the missing element that completes the pattern from six possible choices. This measure has been found to be reliable (α = 0.81−86) for children at this age (Carlson and Jensen, 1981).

#### Wechsler Intelligence Scale for Children—Revised (WISC-R; Wechsler, 1974)

Ten subtests of the WISC-R were administered when children were 8 years of age. Digit span and mazes subtests were not included, and the assessment protocol was 3 h long. From these subtests that were administered, verbal and performance IQ scores were calculated. The Full Scale IQ score was derived from the two subscale scores and used in the analysis.

#### ADHD rating scale (Barkley and Murphy, 1998)

During the young adult visit, ADHD was measured using the ADHD Rating Scale, a self-administered questionnaire comprised of 18 items rated on a four-point scale from 0 (never or rarely) to 3 (very often; α = 0.85) (Barkley and Murphy, 1998). The items in this scale map onto the diagnostic criteria for ADHD, and thus three different scores were derived by summing items from this measure: (1) inattention score, (2) hyperactivity/impulsivity score, and (3) total ADHD score. None of the participants met DSM-IV criteria (i.e., six of nine symptoms must be present to show clinical significance) for either the inattentive or hyperactive/impulsive subtypes of ADHD.

#### Young Adult Self Report (YASR; Achenbach, 1997)

The YASR was completed during the young adult visit. The YASR contains 130 problem items rated as: 0, not true; 1, somewhat or sometimes true; and 2, very true or often true. Based on experts' ratings of the items' consistency with classifications in DSM-IV (A.P.A., 1994), the items were grouped into five DSM-oriented scales (Achenbach et al., 2005): depressive problems (α = 0.88), anxiety problems (α = 0.77), avoidant personality problems (α = 0.76), ADHD problems (α = 0.72) and antisocial personality problems (α = 0.80); and two higher-order scales: internalizing problems (α = 0.93) and externalizing problems (α = 0.85). In addition, the YASR can be scored according to syndrome and problems scales and in order to obtain a better understanding of inattention, we report data from the Attention Problems scale. Including this scale, in addition to the ADHD Rating scale, allows examining the same construct with various measures in order to obtain a better understanding of inattention.

## Results

### Descriptive statistics

In order to examine associations between birth weight group and the variables in the study, a series of *t*-tests comparing ELBW and NBW participants were carried out on measures reflecting demographics and SES (sex, mother's highest level of education, and young adult's highest level of education), as well as the main moderator and outcome variables (fluid intelligence, general intelligence, and scores pertaining to ADHD). Mother's highest level of education was measured according to the following rating scale: 1 = No schooling, 2 = Some primary schooling, 3 = Completed primary school, 4 = Some secondary schooling, 5 = Completed secondary school, 6 = Some community college, 7 = Completed community college, 8 = Some university, 9 = Completed university. Young adult's highest level of education was measured according to the following rating scale: 1 = Less than 7th grade, 2 = Junior high school (9th grade), 3 = Partial high school (10 or 11th grade), 4 = High school graduate, 5 = Partial college (at least 1 year or specialized training), 6 = Standard college or university graduation, 7 = Graduate professional training (MSc, MD, MBA, PhD). Descriptive statistics are presented in Table 1.

**Table 1:** **Means (and *SD*s) on variables of interest by birth weight group**.

	**ELBW**	**NBW**
Sex	54 males; 67 females	56 males; 72 females
Birth weight in grams	841.91 (123.91)^*^	3380.10 (492.63)^*^
Mother's highest level of education	5.61 (1.91)	6.02 (1.98)
Young adult's highest level of education	4.42 (1.33)	4.70 (1.37)
Full scale WISC-R	92.41 (14.66)^*^	103.78 (12.44)^*^
Verbal IQ WISC-R	91.74 (14.23)^*^	101.30 (12.85)^*^
Performance IQ WISC-R	94.83 (16.05)^*^	106.02 (12.90)^*^
General information WISC-R	9.16 (3.07)^*^	10.11 (2.80)^*^
Similarities WISC-R	9.16 (3.38)^*^	10.66 (3.13)^*^
Arithmetic WISC-R	8.12 (2.68)^*^	10.53 (2.66)^*^
Vocabulary WISC-R	8.45 (3.08)^*^	10.01 (2.94)^*^
Comprehension WISC-R	8.63 (2.68)^*^	10.08 (2.42)^*^
Picture completion WISC-R	9.12 (2.68)^*^	11.40 (2.26)^*^
Picture arrangement WISC-R	9.74 (4.12)^*^	11.28 (3.00)^*^
Block design WISC-R	9.21 (3.11)^*^	11.13 (2.96)^*^
Object assembly WISC-R	9.41 (3.06)^*^	10.90 (2.76)^*^
Coding WISC-R	8.71 (3.38)^*^	9.83 (2.65)^*^
RCPM	40.05 (26.53)^*^	55.11 (29.12)^*^
ADHD rating scale inattentive score	3.70 (2.97)	3.06 (2.88)
ADHD rating scale hyperactivity/impulsivity score	4.34 (2.97)	4.38 (2.91)
ADHD rating scale total score	8.04 (5.41)	7.45 (5.10)
YASR attention problems	2.83 (2.35)	2.45 (2.05)

* p < 0.005.

No significant differences were found between the ELBW and NBW groups on sex, mother's highest level of education, and young adult's highest level of education, all *p*s > 0.09. Significant differences were observed between the two groups on birth weight, [*t*_(143.89)_ = −56.43, *p* < 0.0001], fluid intelligence, [*t*_(246.67)_ = −4.27, *p* < 0.0001], general intelligence, [*t*_(235.76)_ = −6.58, *p* < 0.0001], and all WISC-R subtests, all *p*s < 0.005, with NBWs scoring higher on all of these measures. However, ELBW and NBW participants did not significantly differ on ADHD total score, ADHD inattentive score, ADHD hyperactivity/impulsivity score, or YASR attention problems, all *p*s*>* 0.09. Finally, Pearson correlations revealed that the main variables of interest—fluid intelligence and the various ADHD scores—were not related to one another, all *p*s < 0.59. This result suggests that fluid intelligence at age 8 is not simply an early presentation of later ADHD.

### Effects of birth weight group and fluid intelligence on ADHD

In order to examine the moderating role of fluid intelligence in understanding the relation between birth weight group and ADHD symptoms, four separate hierarchical multiple regression analyses were carried out. Each regression included the following outcome variables: ADHD total score, ADHD inattentive score, ADHD hyperactivity/impulsivity score, and YASR attention problems. To reduce multi-collinearity and aid in interpretation, mean centered predictors were used. Next, the interaction terms were computed as the product between birth weight group and the mean-centered measure of fluid intelligence. Given links between fluid intelligence and general intelligence (Embretson, 1995; Engle et al., 1999), general intelligence was included as a covariate in each regression. Thus, the first step of each regression analysis included the main effects of full scale WISC-R, birth weight group, and RCPM score. To test for the moderating effect of fluid intelligence on the link between birth weight group and ADHD symptoms, the interaction product term between birth weight group and RCPM score was entered in the second step. Although the regression models and the terms contained in them were examined for significance, the moderation hypothesis was tested by examining whether the second step significantly increased the variance explained by each model. Interactions were probed and plotted according to guidelines by Aiken and West (1991), such that high and low levels of fluid intelligence were defined as ±1*SD*. Follow-up statistical tests from these probes are reported below.

**Figure 1:**
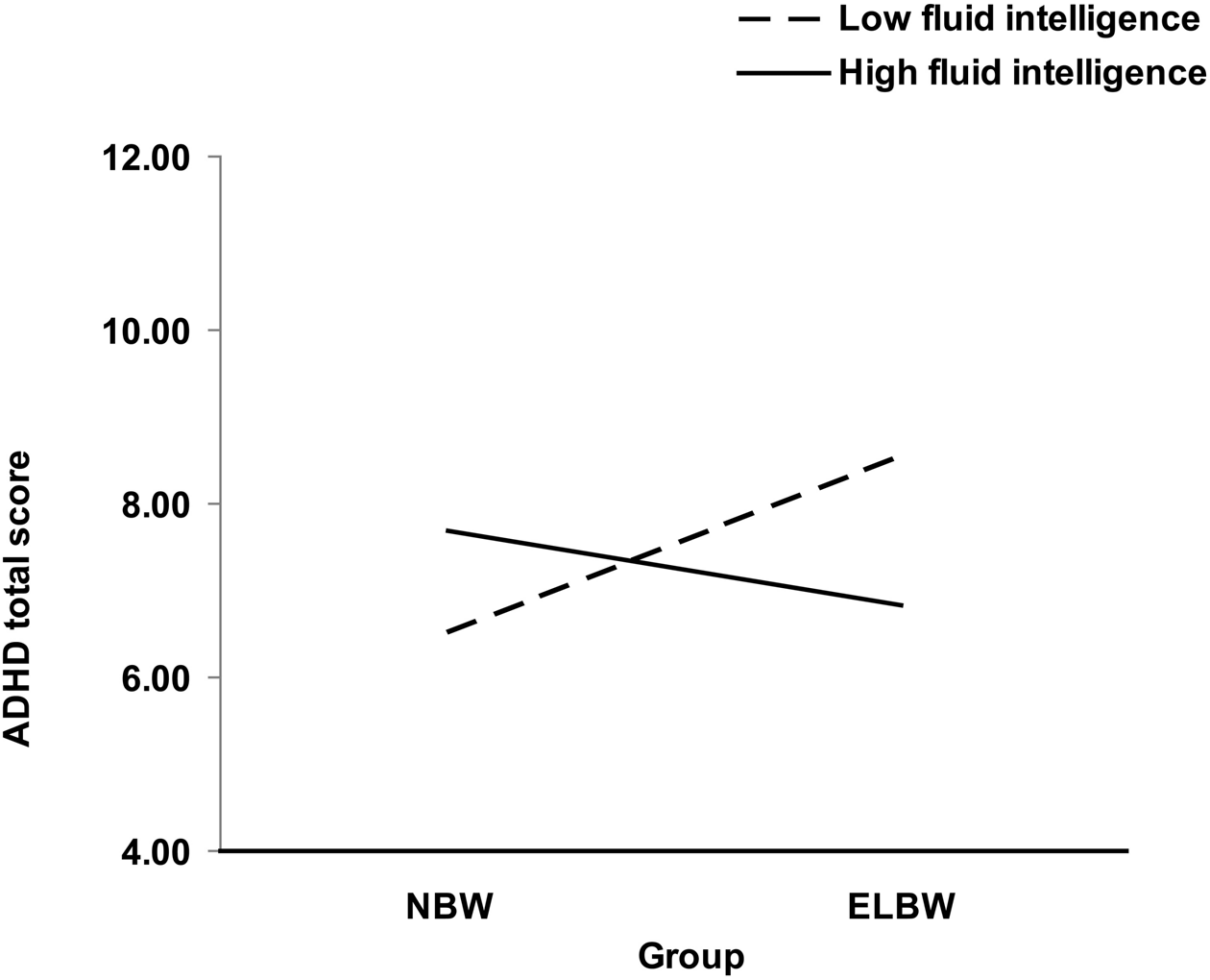
**Joint effect of birth weight group and fluid intelligence at age 8 on ADHD total score in young adulthood**.

For ADHD total score, the interaction between birth weight group and fluid intelligence significantly improved the fit of the model, Δ *R*^2^ = 0.02, [*F*_(1, 244)_ = 4.54, *p* < 0.05] (see Table 2 and Figure 1). To decompose this interaction, follow-up regressions were conducted. The findings indicate that among participants who had low fluid intelligence, birth weight group was related to ADHD score β = 0.20, [*t*_(244)_ = 2.10, *p* < 0.05], such that participants who were at greatest risk at birth (i.e., ELBWs), had the highest ADHD score. However, no such relation emerged in participants with high fluid intelligence, β = −0.09, [*t*_(244)_ = −0.88, *p* = 0.38].

**Table 2:** **Hierarchical multiple regression analysis predicting ADHD symptoms**.

**Variables by step**	**ADHD total score**		**Inattentive score**		**YASR attention problems**	
	***R*^2^**	**β (t)**	***R*^2^**	**β (t)**	***R*^2^**	**β (t)**
Step 1 (df 3/245)	0.004		0.02		−0.004	
WISC-R		−0.004 (−0.04)		−0.09 (−1.01)		0.03 (0.30)
RCPM		0.02 (0.23)		0.05 (0.62)		−0.03 (−0.33)
Birth weight group		0.06 (0.87)		0.09 (1.28)		0.09 (1.33)
Step 2 (df 4/244)	0.02^*^		0.04^*^		0.006	
WISC-R		0.004 (0.05)		−0.08 (−0.91)		0.03 (0.38)
RCPM		0.14 (1.36)		0.19 (1.84)		0.08 (0.75)
Birth weight group		0.06 (0.83)		0.08 (1.24)		0.09 (1.29)
Birth weight group × RCPM		−0.18 (−2.13)^*^		−0.21 (−2.39)^*^		−0.16 (−1.86)

* p < 0.05.

For ADHD inattentive score, the interaction between birth weight group and fluid intelligence significantly improved the fit of the model, Δ *R*^2^ = 0.02, [*F*_(1,244)_ = 5.72, *p* < 0.05] (see Table 2). To decompose this interaction, follow-up regressions were conducted. The findings indicate that among participants who had low fluid intelligence, birth weight group was related to ADHD inattentive score β = 0.24, [*t*_(244)_ = 2.58, *p* < 0.01], such that participants who were at greatest risk at birth (i.e., ELBWs), had the highest ADHD inattentive score. However, no such relation emerged in participants with high fluid intelligence, β = −0.07, [*t*_(244)_ = −0.76, *p* = 0.45].

When predicting YASR attention problems a trend was found for the interaction between birth weight group and fluid intelligence, Δ *R*^2^ = 0.01, [*F*_(1, 244)_ = 3.46, *p* = 0.06] (see Table 2). To decompose this interaction, follow-up regressions were conducted. The findings indicate that among participants who had low fluid intelligence, birth weight group was related to YASR attention problems β = 0.21, [*t*_(244)_ = 2.24, *p* < 0.05], such that participants who were at greatest risk at birth (i.e., ELBWs), had the most YASR attention problems. However, no such relation emerged in participants with high fluid intelligence, β = −0.04, [*t*_(244)_ = −0.36, *p* = 0.71].

Finally, the regression model was not significant for ADHD hyperactivity/impulsivity score, Δ *R*^2^ = 0.01, [*F*_(1, 244)_ = 2.06, *p* = 0.15].

## Discussion

This longitudinal study prospectively followed a cohort of ELBW survivors and a matched NBW control sample at age 8 and again during young adulthood (22–26). During the 8-year visit, participants completed measures of fluid and general intelligence. Approximately 15 years later, as young adults, participants provided self-report of ADHD symptoms. As predicted, fluid intelligence moderated the link between birth weight and ADHD symptoms. In particular, ELBW survivors with poor fluid intelligence were at the greatest risk for later ADHD symptoms, particularly symptoms pertaining to the inattentive sub type of ADHD. These findings suggest that fluid intelligence is an important mechanism involved in developmental trajectories that lead ELBW survivors to develop later symptoms of ADHD.

Importantly, our analyses for ADHD total score and ADHD inattentive score were statistically significant even when general intelligence was included in the models as a covariate. This result suggests that fluid intelligence plays a specific role in the association between birth weight group and ADHD above and beyond the role of general intelligence. This finding is important given the presence of substantial correlations between fluid intelligence and measures of general intelligence (e.g., Embretson, 1995; Engle et al., 1999).

Our findings are also consistent with work on VLBW participants, suggesting that links between extreme preterm birth and inattention was mediated by working memory, a specific type of EF (Nadeau et al., 2001). Our findings extend this previous research in two important ways: (1) using a moderation approach, we extended this work to ELBW survivors, and (2) our longitudinal study extended over a longer period of time, such that birth weight and fluid intelligence at age 8 interacted to predict ADHD symptoms in young adulthood. Therefore, our findings make a major contribution to research examining developmental trajectories leading to negative outcomes among survivors born at the most severe levels of early adversity.

The present study also extends previous work by Boyle et al. (2011) who found no evidence of group differences in ADHD symptoms during young adulthood with the same cohort of ELBW survivors and controls. This finding was replicated in the present study when directly comparing the two groups. However, the hierarchical regressions revealed that the link between birth weight group and ADHD is more complex, with an interaction between birth weight group and fluid intelligence in predicting later ADHD symptoms, and symptoms of inattention in particular. It should be noted that the interaction between birth weight group and fluid intelligence explained only a small amount of the variance in the ADHD variables examined. This suggests that there are other factors involved in adult ADHD symptoms that were not measured in the present study.

Several authors have proposed that symptoms of ADHD arise from a primary deficit in EF (Pennington and Ozonoff, 1996; Barkley, 1997; Schachar et al., 2000; Castellanos and Tannock, 2002), or that poor EF is an earlier presentation of ADHD. However, in the present study, we did not find a direct relation between fluid intelligence at age 8 and ADHD symptoms during young adulthood. This finding is in line with Willcutt et al.'s (2005) argument that difficulties with EF appear to be only one of many important components of the complex neuropsychology of ADHD.

In the present study, fluid intelligence moderated ADHD symptoms using the total ADHD score on the ADHD Rating Scale (Barkley and Murphy, 1998), as well as the inattentive score on this scale. Furthermore, we observed a trend for Attention Problems using the YASR. These findings are consistent with previous research suggesting associations between birth weight and ADHD (Botting et al., 1997; Indredavik et al., 2004; Hack et al., 2009; Johnson et al., 2010), as well as EF and ADHD (Chhabildas et al., 2001; Nigg et al., 2005; Willcutt et al., 2005), particularly for the inattentive ADHD subtype. Our findings suggest that it is the combination of *both* being born at ELBW *and* having poor fluid intelligence that together contribute to the prediction of later ADHD symptoms, and particularly symptoms of inattention.

It is important to note that the RCPM is only one of many fluid intelligence measures. In addition, although fluid intelligence and EF involve the same underlying processes (Blair, 2006), there is some debate about equating these two constructs (Birney et al., 2006; Burgess et al., 2006; Garlick and Sejnowski, 2006; Heitz et al., 2006). For example, some argue that working memory and fluid intelligence are highly related but separable, and suggest that the mechanism behind the relation is controlled attention—an ability that is dependent on normal functioning of the prefrontal cortex (Heitz et al., 2006).

In summary, the present study followed prospectively the oldest known cohort of ELBW survivors and a matched control sample over a period of 26 years. Fluid intelligence was assessed at 8 years of age, and ADHD symptoms were assessed at 22 to 26 years of age. Our findings indicate that among individuals with poor fluid intelligence measured at age 8, ELBW survivors had the highest level of ADHD symptoms as young adults. These findings point to the importance of examining possible moderating mechanisms that contribute to developmental outcomes and risk for psychopathology.

### Conflict of interest statement

The authors declare that the research was conducted in the absence of any commercial or financial relationships that could be construed as a potential conflict of interest.
